# Supplementary material for: Monitoring sexual hormones in women going to high altitude—a pilot study
Source: Front Glob Womens Health. 2025 Mar 31;6:1544832. doi: 10.3389/fgwh.2025.1544832 (PMC11994732; doi:10.3389/fgwh.2025.1544832)
Supplement: Supplementary file 1 [file Table1.pdf]

Supplement to:

Monitoring sexual hormones in women going to high altitude – a pilot study

Aijan Taalaibekova,[1] Michelle Meyer,[2*] Stefanie Ulrich,[2] Gulzada Mirzalieva,[1] Maamed Mademilov,[1] Mona Lichtblau,[2] Cornelia Betschart,[3] Talant M. Sooronbaev,[1] Silvia Ulrich [2] Konrad E. Bloch,[2] Michael Furian[2,4]*

[1] *National Center of Cardiology and Internal Medicine, Bishkek, Kyrgyzstan*

[2] *Pulmonology Department, University Hospital Zurich, Zurich, Switzerland*

[3] *Gynaecology Department, University Hospital Zurich, Zurich, Switzerland*

[4] *Research Department, Swiss University of Traditional Chinese Medicine, Bad Zurzach, Switzerland*

[*] *Shared first authorship*

Funding

This study was partly funded by the Swiss National Sciences Foundation (210361).

Correspondence

Taalaibekova Aijan

National Center of Cardiology and Internal Medicine,

Bishkek, Kyrgyzstan

Aijan_taalaibekova@bk.ru

Supplement Table S1: Nocturnal pulse oximetry

**Variable**	**760 m**	**1st night at 3,600 m**	**2nd night at 3,600 m**
Nocturnal SpO_2, %	95.5 ± 0.6	79.3 ± 0.6*	82.4 ± 0.6*#
Heart rate, bpm	65 ± 2	85 ± 2*	79 ± 2*#
Oxygen desaturation index, events/hour	0.9 ± 0.5	8.6 ± 2.6*	6.5 ± 0.6*#
SpO_2 <95%, % TIB	15 ± 2	100 ± 2*	100 ± 2*
SpO_2 <90%, % TIB	1 ± 1	99 ± 1*	98 ± 1*
SpO_2 <85%, % TIB	0 ± 4	94 ± 4*	69 ± 4*#
SpO_2 <80%, % TIB	0 ± 5	48 ± 6*	20 ± 6*#
SpO_2 <75%, % TIB	0 ± 4	13 ± 4*	5 ± 4
SpO_2 <70%, % TIB	0 ± 1	3 ± 1	0 ± 1

Values presented as means ± SE. *$p<0.05$ compared to 760 m; #$p<0.05$ 2nd night versus 1st night at 3,600 m. SpO_2, arterial oxygenation assessed by finger pulse oximetry; TIB, time in bed.

Supplement Table S2. The effect of sex hormones and altitude on arterial oxygenation – a mixed linear regression model

Dependent variable: Daytime arterial oxygenation by finger pulse oximetry, %	**Coefficient**	**Standardized beta coefficient**	**Std. Error**	**P-value**	**95% CI**
**Progesterone concentration, mcg/ml**	0.17	0.21	0.04	<0.001	0.09 to 0.25
**Estrogen, ng/ml**	-0.01	-0.13	0.00	0.007	-0.01 to 0.00
**Luteinizing hormone, mIU/ml**	0.00	0.00	0.02	0.962	-0.03 to 0.03
**Altitude of assessment**					
760 m (*reference*)	96.1	NA	0.4	<0.001	95.3 to 96.8
Difference between 3,100 and 760 m	-3.3	-0.32	0.5	<0.001	-4.3 to -2.2
Difference between 3,600 and 760 m	-9.8	-0.95	0.6	<0.001	-10.9 to -8.6

Mixed linear regression analysis including all available measurements. Due to the small sample size, the parameters for the mixed models were predefined. For example, according to this model, a 1 mcg/ml increase in progesterone is associated with a 0.17% increase in SpO_2, independent of altitude and other hormones; an altitude sojourn at 3,600 m is associated with a 9.8% decrease in SpO_2, independent of the hormone levels.
